# Keel bone damage and access to functional resources and housing areas in laying hens: a systematic thematic review

**DOI:** 10.3389/fvets.2026.1950640

**Published:** 2026-10-01

**Authors:** Serge Alindekon, Stefanie Petow, Helen Louton

**Affiliations:** Department of Veterinary Sciences, Faculty of Veterinary Medicine, LMU Munich, Munich, Germany

**Keywords:** housing areas, keel bone damage, laying hens, resource access, welfare

## Abstract

Keel bone damage (KBD) is a major welfare concern in laying hens; however, evidence on its relationship with access to and use of functional resources and housing areas has not been systematically synthesised. This review examined associations between KBD and perches, resting locations, nests, feeders, drinkers, litter and floor areas, outdoor ranges, popholes, wintergardens, and other defined housing areas. PubMed and Web of Science were searched for empirical studies published between 2006 and 2026 involving laying hens, assessing KBD, and reporting relevant resource- or area-related outcomes. Of 345 records identified, 233 remained after deduplication. Following title and abstract screening, 50 reports underwent full-text assessment, of which 17 were excluded; one additional study was identified manually, yielding 34 included studies. Owing to substantial variation among housing systems, KBD assessment methods, outcome measures, and temporal designs, the findings were synthesised narratively by resource category and direction of association. The evidence base was limited and unevenly distributed, with most studies focusing on perches, elevated structures, and resting locations. Hens with keel fractures often accessed elevated resources less frequently, descended more slowly, or made fewer vertical transitions. However, reduced access did not always correspond to reduced occupancy, as some affected hens remained longer on perches or in nests once these locations had been reached. Evidence for feeders, drinkers, outdoor areas, and ground-level resources was sparse and inconsistent. Few studies established whether changes in resource use preceded or followed KBD, limiting causal interpretation. Overall, KBD was most consistently associated with the use of physically demanding elevated resources, while evidence for other functional areas remained uncertain. Resource occupancy should not be interpreted as unrestricted access because affected hens may reach a resource less often yet remain there longer. Future studies should use longitudinal individual-level designs, distinguish access from occupancy and departure, and combine automated tracking with direct behavioural observation.

## 1 Introduction

Keel bone damage (KBD) is a widespread and persistent welfare concern in commercial laying hens. The term commonly encompasses fractures and deviations, which may coexist but should not be considered biologically interchangeable because they generally arise through different processes and may have different welfare consequences (1, 2). KBD has a multifactorial origin involving bird-related susceptibility and mechanical conditions within the housing environment (3, 4). Fractures may develop when forces associated with flight, landing, collisions, or other loading events act on a keel whose structural strength is influenced by skeletal development, genetic background, nutrition, and the mineral demands associated with egg production (2–4). In contrast, deviations are generally thought to develop gradually through bone remodelling in response to prolonged pressure during perching (2, 5, 6).

Keel abnormalities have been described in poultry since at least the beginning of the twentieth century (e.g. (7),), indicating that the condition is not a recent consequence of changes in laying-hen housing. KBD has since been documented in conventional and furnished cages, single-tier systems, aviaries, and free-range systems, although reported prevalence varies with housing system, age, genetic strain, study population, and assessment method (2, 3, 8). In an end-of-lay examination of 4,794 hens from 40 Danish flocks, for example, keel bone fracture prevalence ranged from 53 to 100% in non-cage systems and from 50 to 98% in enriched cages (9).

The functional consequences of KBD are particularly relevant in structurally complex housing. The keel provides the attachment surface for the major flight muscles, and hens rely on coordinated wing and hindlimb movements during flight, landing, and wing-assisted locomotion between levels (10, 11). Keel bone fractures are also associated with pain and impaired mobility, raising concern that affected hens may have difficulty navigating their environment (12, 13). At the same time, longer distances and steeper transition angles, especially during downward movements, increase mechanical demands and may make transitions more difficult to complete without balancing movements, falls, or collisions (14).

These concerns have become increasingly important as laying-hen production has shifted away from conventional cages toward more structurally complex systems. Furnished cages, multi-tier aviaries, free-range systems, and organic systems are intended to provide greater opportunities for species-specific and highly motivated behaviours. Depending on the system, hens may perch, nest, dust-bathe, forage, explore litter, move between levels, and access verandas, wintergardens, or outdoor ranges (15–18). These opportunities are valuable only when hens can reach, use, and leave the resources and functional areas provided. The same features may also increase physical demands through repeated vertical transitions, elevated structures, movement between functional areas, and exposure to falls, collisions, or unstable landings.

The relationship between KBD and resource use may therefore operate in both directions. Pain, mechanical impairment, reduced mobility, or behavioural compensation may alter how affected hens access, occupy, or move between feeding, drinking, nesting, perching, resting, litter, and outdoor areas. Studies of perch access, aviary-zone occupancy, and outdoor use suggest that keel bone fractures can be associated with changes in how hens interact with particular parts of their environment (8, 13, 19, 20). Conversely, the design, positioning, and use of resources may influence movement patterns, mechanical loading, and exposure to falls or collisions. In this context, mechanical loading refers to the forces transmitted to the keel during perching, take-off, landing, or collisions; their magnitude and frequency can be affected by perch design, transition distance and angle, and contact with housing structures (1, 5, 14, 21). Patterns of resource access and use may therefore reflect the functional consequences of KBD while also providing information about environmental conditions that contribute to its development.

This relationship has implications for both hens and farmers. For hens, impaired or delayed access may reduce comfort, restrict behavioural choice, and make valued or biologically necessary resources more difficult to reach. Experimental analgesia studies provide evidence that keel bone fractures are painful (12), while severe fractures have also been associated with a more persistent negative affective state (22). Changes in perch, nest, litter, feeder, drinker, or range use may therefore indicate more than differences in location. They may reflect pain, impaired locomotor capacity, behavioural compensation, or difficulty reaching resources supporting highly motivated behaviours. For farmers, reduced access to feeders and drinkers may impair feed and water intake, with potential consequences for health, mortality, and egg production. Restricted access to nest boxes may also increase floor laying, thereby increasing the risk of dirty, damaged, or lost eggs and creating additional management demands (23, 24). More broadly, altered occupancy of functional areas may indicate that the resources provided are not equally usable by all hens. A central question therefore emerges: do hens with KBD fully benefit from the resources available within the housing system, or does the damage restrict or otherwise alter their ability to access and use them?

Previous reviews have synthesised evidence on KBD prevalence, diagnosis, risk factors, housing-system associations, pain, broader welfare consequences, and preventive measures (e.g. (2–4, 8),). However, they have not been organised primarily around hens’ access to and use of specific resources and functional areas. Relevant evidence remains distributed across studies of perching, nesting, aviary-zone occupancy, litter use, vertical transitions, pophole passage, and outdoor ranging. These studies also differ in whether resource use is examined as a possible consequence of KBD, a potential contributor to damage, or an associated behavioural or spatial outcome. It therefore remains unclear which resources are most consistently affected, whether KBD restricts access or alters occupancy and movement, and whether associations differ among elevated, ground-level, essential, and outdoor resources.

To clarify the terminology used in this review, a resource refers to a housing feature or substrate that hens can access or use, while a functional resource refers specifically to one supporting a biological or behavioural function. A functional area refers to a spatially defined part of the housing environment supporting one or more such functions, whereas housing area is used more broadly for defined spatial zones. These categories may overlap, as a housing area can contain several functional resources and support several functions.

This systematic thematic review synthesises evidence on the relationship between KBD and resource access and use in laying hens. It considers perch use and resting location; nest, feeder, and drinker access or use; litter and other ground-level areas; and outdoor ranges, verandas, wintergardens, and popholes. The review distinguishes studies examining KBD as a potential influence on resource access or use from those examining resource use, resource design, or housing layout as potential contributors to KBD. By organising the evidence by resource category and direction of association, the review aims to clarify how KBD relates to hens’ functional interaction with the housing environment and to identify implications for housing design, welfare assessment, management, and future research.

## 2 Materials and methods

### 2.1 Literature search and study identification

This systematic thematic review examined the relationship between KBD and the use of space and functional resources by laying hens. The literature search was conducted in PubMed and the Web of Science Core Collection between April and May 2026. The search covered publications from 2006 to 2026, with no restriction on publication type during the initial database search.

The search strategy combined three groups of terms relating to (1) laying hens; (2) KBD; and (3) resource use, resource access, resource visits, or spatial use. Terms within each group were combined using the Boolean operator OR, and the three groups were combined using AND. Database-specific syntax was applied.

The following search query was used in Web of Science:

TS = ((“laying hen*” OR “laying chicken*” OR “layer hen*” OR “layer chicken*” OR “commercial layer*” OR “*Gallus gallus*” OR “*Gallus gallus* domesticus”) AND ((keel NEAR/3 bone*) OR (keel NEAR/3 fracture*) OR (keel NEAR/3 damage*) OR (keel NEAR/3 injur*) OR (keel NEAR/3 lesion*) OR (keel NEAR/3 deform*) OR (keel NEAR/3 deviation*) OR “keel bone damage” OR “keel bone fracture*” OR “keel bone deviation*” OR “keel fracture*” OR “keel deviation*” OR (stern* NEAR/3 fracture*) OR (stern* NEAR/3 damage*) OR (stern* NEAR/3 injur*) OR (stern* NEAR/3 lesion*) OR (stern* NEAR/3 deform*) OR (stern* NEAR/3 deviation*)) AND (“resource use” OR “resource access” OR “resource utilisation” OR “resource utilization” OR “resource occupancy” OR “resource preference” OR “space use” OR “spatial use” OR “spatial distribution” OR “location use” OR “animal location*” OR location* OR position* OR zone* OR tier* OR “top tier” OR “middle tier” OR “lower tier” OR litter OR “litter area” OR floor OR “floor area” OR nest* OR “nest box*” OR perch* OR roost* OR aviary OR “multi-tier” OR “multi tier” OR “free range” OR “range use” OR “outdoor range” OR veranda OR wintergarden OR “winter garden” OR pophole* OR “pop hole*” OR feeder* OR drinker* OR “feeding behaviour” OR “feeding behavior” OR “drinking behaviour” OR “drinking behavior” OR “feed intake” OR “water intake” OR “feeding time” OR “drinking time” OR “feeding activity” OR “drinking activity” OR “feed consumption” OR “water consumption” OR (feed* NEAR/3 (use OR access OR intake OR behaviour OR behavior OR activity OR time OR station* OR trough*)) OR (water* NEAR/3 (use OR access OR intake OR consumption OR behaviour OR behavior OR activity)) OR (drink* NEAR/3 (use OR access OR behaviour OR behavior OR activity OR time)) OR RFID OR “radio frequency identification” OR “ultra-wideband” OR UWB OR tracking OR sensor*)).

The following search query was used in PubMed:

((“laying hen*” OR “laying chicken*” OR “layer hen*” OR “layer chicken*” OR “commercial layer*” OR “*Gallus gallus*” OR “*Gallus gallus* domesticus”) AND ((“keel” AND (“bone*” OR “fracture*” OR “damage*” OR “injur*” OR “lesion*” OR “deform*” OR “deviation*”)) OR “keel bone damage” OR “keel bone fracture*” OR “keel bone deviation*” OR “keel fracture*” OR “keel deviation*” OR (“stern*” AND (“fracture*” OR “damage*” OR “injur*” OR “lesion*” OR “deform*” OR “deviation*”))) AND (“resource use” OR “resource access” OR “resource utilisation” OR “resource utilization” OR “resource occupancy” OR “resource preference” OR “space use” OR “spatial use” OR “spatial distribution” OR “location use” OR “animal location*” OR “location*” OR “position*” OR “zone*” OR “tier*” OR “top tier” OR “middle tier” OR “lower tier” OR “litter” OR “litter area” OR “floor” OR “floor area” OR “nest*” OR “nest box*” OR “perch*” OR “roost*” OR “aviary” OR “multi-tier” OR “multi tier” OR “free range” OR “range use” OR “outdoor range” OR “veranda” OR “wintergarden” OR “winter garden” OR “pophole*” OR “pop hole*” OR “feeder*” OR “drinker*” OR “feeding behaviour” OR “feeding behavior” OR “drinking behaviour” OR “drinking behavior” OR “feed intake” OR “water intake” OR “feeding time” OR “drinking time” OR “feeding activity” OR “drinking activity” OR “feed consumption” OR “water consumption” OR (“feed*” AND (“use” OR “access” OR “intake” OR “behaviour” OR “behavior” OR “activity” OR “time” OR “station*” OR “trough*”)) OR (“water*” AND (“use” OR “access” OR “intake” OR “consumption” OR “behaviour” OR “behavior”)))).

### 2.2 Record merging and duplicate removal

All records retrieved from PubMed and Web of Science were exported, combined into a single reference library, and imported into Rayyan web application (https://www.rayyan.ai) for record management and screening.

Potential duplicates were identified using Rayyan’s duplicate-detection function and were manually verified using the title, authors, publication year, journal, DOI, and PubMed identifier, when available. Confirmed duplicates were removed before title and abstract screening.

### 2.3 Eligibility assessment and study selection

Titles and abstracts were screened by one reviewer (SA) using the manual screening function of the Rayyan. Records were excluded when they clearly did not concern domestic laying hens, KBD, or the use of a functional resource or defined housing area. Records meeting these broad criteria, or for which eligibility remained uncertain, were retained for full-text assessment.

The same reviewer assessed the retained full texts against the predefined inclusion and exclusion criteria presented in Table 1 and made the final inclusion decisions. The criteria were applied through a structured eligibility assessment to support consistency throughout the selection process.

**Table 1:** Inclusion and exclusion criteria.

Criterion	Inclusion criteria	Exclusion criteria
Population	Domestic laying hens (*Gallus gallus domesticus*)	Other species, or studies in which laying-hen data could not be separated
Study type	Original empirical research	Reviews, meta-analyses, editorials, commentaries, protocols, book chapters, and conference abstracts
Publication period	Published between 2006 and 2026	Published before 2006
Language	Full text available in English	Full text not available in English
KBD assessment	Presence, occurrence, type, location, or severity of keel bone fractures, deviations, deformities, lesions, or other clearly defined keel or sternal damage, assessed using palpation, imaging, visual examination, post-mortem examination, dissection, or another described method	KBD not assessed, insufficiently defined, or not reported separately from other skeletal conditions
Eligible resource or housing area	Perches and roosts; nests and nest boxes; feeders; drinkers; litter and floor areas; outdoor ranges, verandas, wintergardens, and popholes; aviary tiers, zones, or other defined functional areas	No specific resource or defined housing area examined
Eligible resource-use or spatial outcome	Number or frequency of visits; duration or proportion of time spent; occupancy; access; latency; preference; location; spatial distribution; transitions; or movement between defined housing areas	General behaviour, activity, gait, locomotor ability, or movement not linked to a specific resource or defined housing area
Relationship examined	Statistical or clearly reported empirical relationship between KBD and an eligible resource-use, spatial-use, resource-design, or housing-layout variable	KBD and resource use reported separately without examination of a relationship between them

An included study could be assigned to more than one resource or housing-area category when it reported multiple eligible resources, areas, or outcomes. The numbers of records identified, screened, assessed in full text, excluded, and included are presented in the PRISMA flow diagram in the Results section.

### 2.4 Data extraction and thematic classification

Data were extracted manually from the full-text reports into a structured Microsoft Excel 365 spreadsheet organised around general study characteristics and the relevant resource or housing-area category (Table 2). Studies reporting more than one category were coded separately for each relevant outcome.

**Table 2:** Information extracted from the included studies.

Extraction category	Information collected
Bibliographic information	Authors, publication year, and country
Study characteristics	Study objective, study design, and experimental or commercial setting
Population	Hen strain, age, stage of lay, number of hens, and number of groups or flocks
Housing system	Housing type, flock size, system configuration, and relevant resource characteristics
KBD assessment	KBD definition, fracture or deviation type, severity, assessment method, and timing
Resource category	Perch, roost, nest, feeder, drinker, litter, floor, range, veranda, wintergarden, pophole, tier, zone, or other defined housing area
Resource-use outcome	Visits, visit frequency, duration, proportion of time spent, occupancy, access, latency, preference, location, spatial distribution, or transitions
Perch and roost use	Perch or roost visits, occupancy, duration of use, height or location, night-time use, and transitions
Nest use	Nest visits, occupancy, duration, nest preference, laying location, and transitions
Feeder use	Feeder visits, visit frequency, duration, feeding time, access, and feed intake, when reported
Drinker use	Drinker visits, visit frequency, duration, drinking time, access, and water intake, when reported
Litter and floor use	Time spent on the litter or floor, occupancy, visits, location, and movement between areas
Outdoor and connected areas	Range, veranda, or wintergarden visits; duration of use; exits and entries; latency; distance travelled; and pophole use
Tier and spatial distribution	Tier or zone occupancy, vertical distribution, location, transitions, and movement between housing levels
Measurement method	Direct observation, video recording, RFID, ultra-wideband tracking, sensors, or other methods
Direction of the relationship	KBD examined in relation to resource use, or resource use, resource design, or housing layout examined in relation to KBD
Statistical information	Statistical models, effect estimates, measures of uncertainty, and significance
Main findings	Direction, magnitude, and interpretation of the reported relationship

Extracted findings were organised thematically by resource or housing-area category and according to whether KBD was examined as a potential influence on resource use or whether resource use, resource design, or housing layout was examined as a potential contributor to KBD. Study characteristics were summarised descriptively using counts and percentages for categorical variables and the median and range for individual-hen analytical sample sizes. Graphical summaries were produced in Python 3.11.14 using pandas 3.0.3 and Matplotlib 3.10.7.

Given the heterogeneity in housing systems, study designs, KBD assessments, resource-use measurements, and statistical analyses, no pooled effect estimates were calculated, and the findings were synthesised narratively.

## 3 Results

### 3.1 Study selection

The searches identified 345 records, including 220 from Web of Science and 125 from PubMed. After 112 duplicates were removed, 233 records underwent title and abstract screening. Of these, 183 were excluded because one or more core eligibility elements were clearly absent, leaving 50 database reports for full-text assessment. Manual searching identified one additional report, bringing the total number of full-text reports assessed to 51.

Seventeen full-text reports were excluded. Reports were excluded when KBD was absent or insufficiently defined, or when no eligible resource or housing-area outcome or relevant KBD-resource relationship was reported. Other reasons included a focus limited to general behaviour or locomotion, an ineligible publication type, duplication or overlap with another report, or insufficient usable information. The remaining 34 unique primary studies were included in the systematic thematic review (Figure 1).

**Figure 1:**
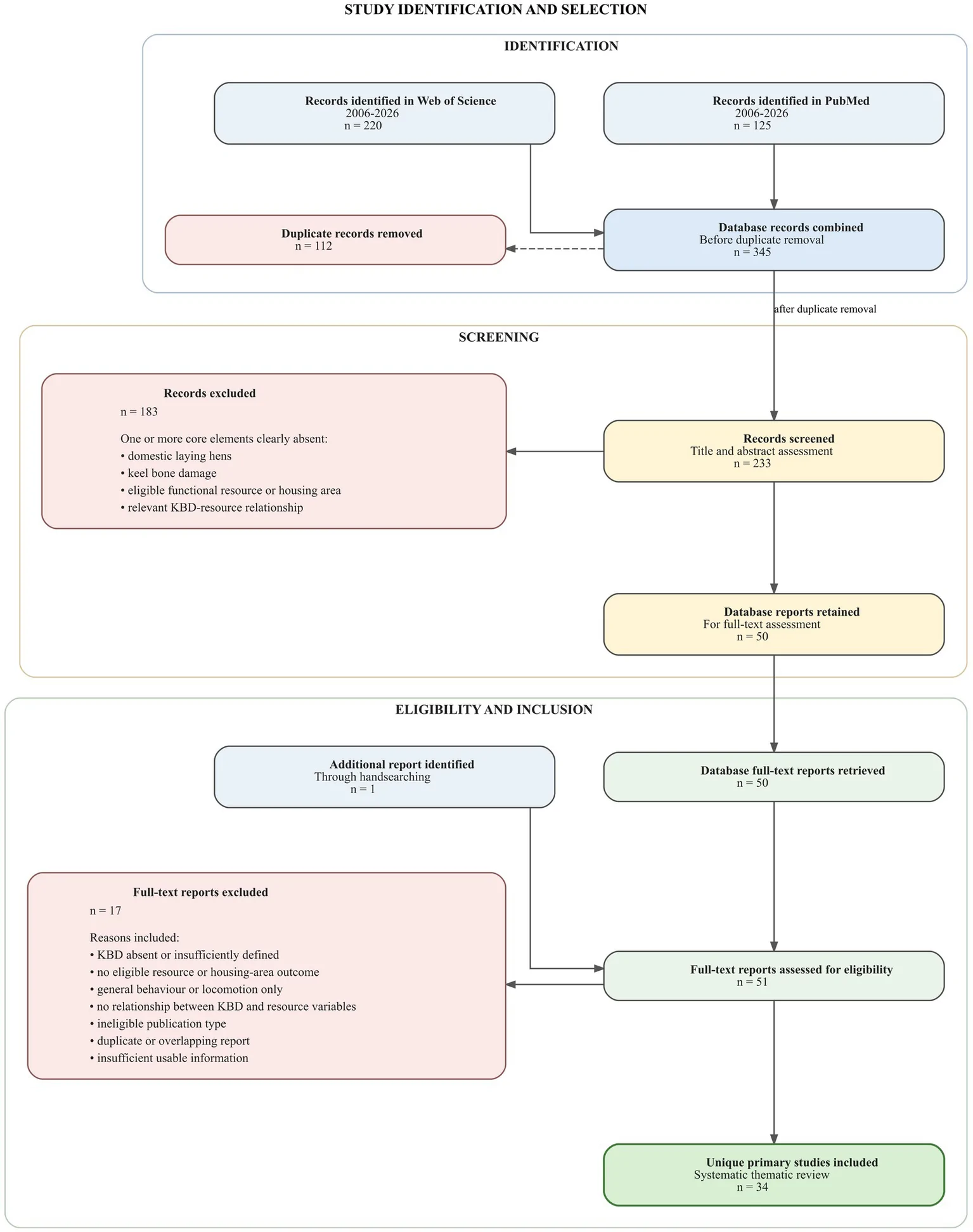
PRISMA-style flow diagram of study identification, screening, eligibility assessment, and inclusion.

### 3.2 Characteristics of the evidence base

The evidence base comprised 34 primary studies examining KBD in relation to hens’ access to, occupancy of, and use of functional resources and housing areas (Figure 2). Non-cage floor or pen systems were the most represented housing type, accounting for 13 studies (38.2%), compared with eight studies conducted in multi-tier aviaries and five in furnished or enriched cages (Figure 2A). Other housing contexts were represented by relatively few studies.

**Figure 2:**
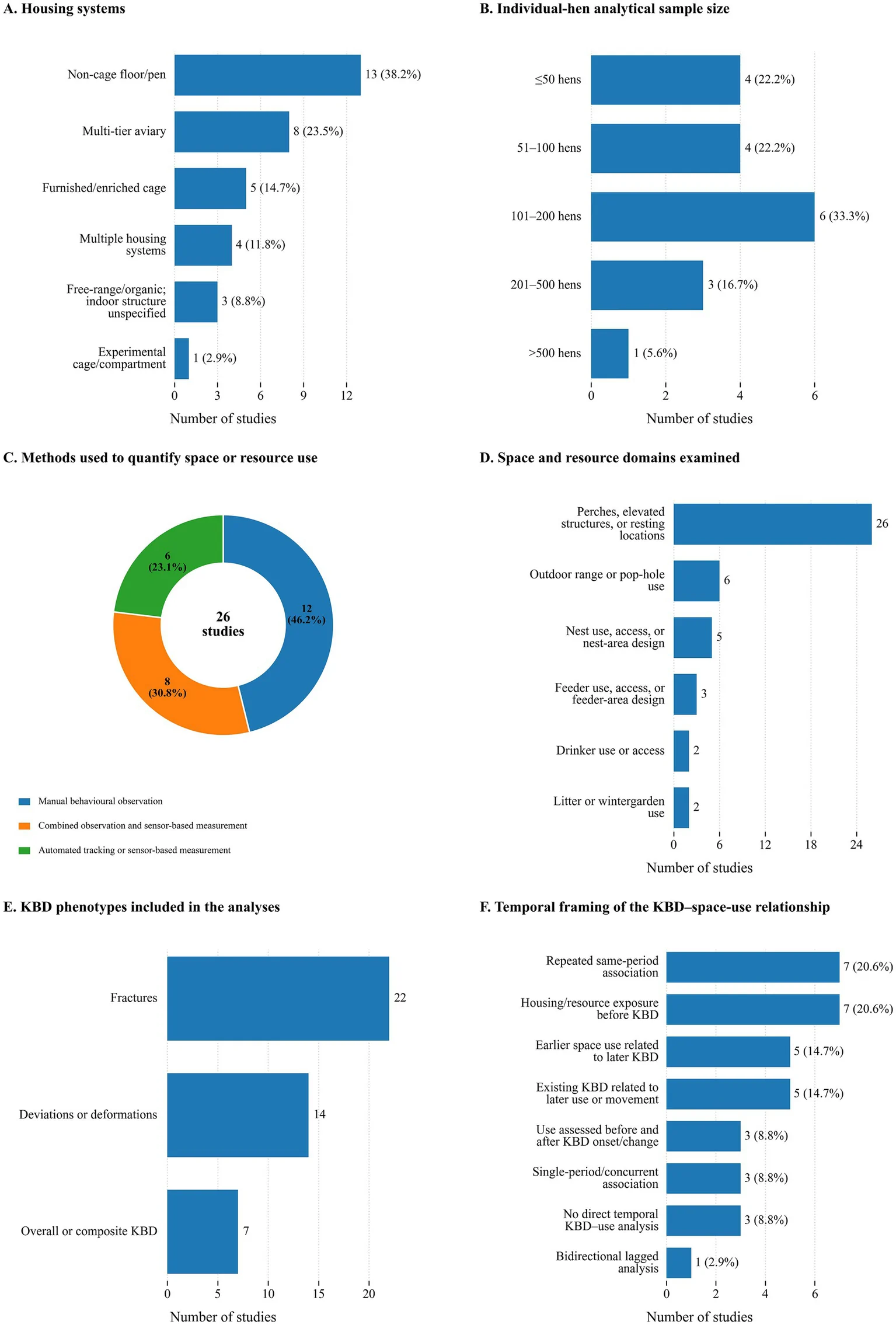
Characteristics of the evidence base examining KBD in relation to access to, occupancy of, and use of functional resources and housing areas in laying hens. Panels summarise the included studies according to **(A)** housing systems, **(B)** individual-hen analytical sample size, **(C)** methods used to quantify space or resource use, **(D)** space and resource domains examined, **(E)** KBD phenotypes included in the analyses, and **(F)** temporal framing of the KBD-space-use relationship. In Panels D and E, categories were not mutually exclusive because individual studies could examine several space or resource domains and include more than one KBD phenotype in their analyses, respectively. Consequently, the totals across categories may exceed the number of included studies. KBD, keel bone damage.

Only 18 studies directly linked individual-hen KBD status with resource- or area-use outcomes. Among these studies, analytical sample sizes ranged from 20 to 601 hens, with a median of 110.5 hens. Most included no more than 200 hens, and only four exceeded this threshold (Figure 2B).

Among the 26 studies that directly quantified hens’ access to, occupancy of, or use of housing areas and resources, manual behavioural observation remained the most common approach. It was used alone in 12 studies, twice as often as automated or sensor-based measurement alone, which was used in six studies. A further eight studies combined observational and sensor-based methods (Figure 2C).

The evidence was strongly centred on perches and other elevated structures. These were examined in 26 studies, compared with six studies examining outdoor range or pop-hole use, the next most frequent domain. Nest, feeder, drinker, and litter or wintergarden outcomes were comparatively uncommon (Figure 2D). Because individual studies could examine more than one domain, these categories were not mutually exclusive.

Fractures were examined in 22 of the 31 studies that analysed at least one KBD phenotype. Deviations or deformations were examined in 14 studies, whereas seven used an overall or composite KBD measure (Figure 2E). These categories were also not mutually exclusive because some studies analysed more than one phenotype.

Direct temporal ordering between KBD and resource or area use was established in fewer than half of the studies (14/34, 41.2%). Five studies related earlier resource or area use to later KBD, five examined subsequent use patterns in hens with existing KBD, three evaluated use before and after an identified change in KBD status, and only one tested bidirectional lagged relationships. The remaining 20 studies relied on concurrent or same-period associations, housing or resource exposure, or designs that did not establish whether altered use preceded or followed KBD (Figure 2F).

### 3.3 Perch use and resting location

Studies concerning perches and resting locations examined both directions of the relationship, namely, whether KBD influenced perch access and use, and whether perch use or design potentially contributed to KBD (Table 3).

**Table 3:** Perch use and resting-location outcomes associated with KBD.

Study	Housing, birds, and KBD assessment	Outcome variable	Key findings	Evidence pattern
Wei et al. (26)	Individually housed furnished cages; Lohmann White hens; 90 hens; 17–42 weeks; KBD assessed by palpation and dissection, with hens classified as normal keel, deviated keel, or fractured keel.	Perching and jumping behaviours.	Fractured-keel hens perched less than normal-keel hens: 10.03 ± 0.46% vs. 13.07 ± 0.99% of observations (p = 0.015). Jumping was also significantly lower in fractured-keel hens (p < 0.05; exact value not reported).	KBD reduced perch use and perch-related mobility.
Rentsch et al. (19)	Experimental non-cage pens; Brown Nick and Nick Chick hens; 80 hens; 26–39 weeks; KBF assessed by radiography and classified by fracture state, including visible fracture gap.	Perch or elevated nest-box access, defined as vertical locomotion; walking pace on ramps leading to perches.	Odds of vertical locomotion, including perch or nest access, decreased when a visible fracture gap was present (p = 0.004). Walking pace on ramps was not affected by fractures (p = 0.29).	KBD reduced perch/elevated-resource access.
Nasr et al. (12)	Floor pen with raised perches; Lohmann Brown hens; 58 hens; 35 weeks; KBF assessed by palpation and dissection.	Latency to fly down from elevated perches.	Hens with fractures took 6 times longer to fly down from 100-cm perches than hens without fractures: 37.0 ± 9.3 s vs. 6.6 ± 11.4 s (p = 0.04). They took 8 times longer from 150-cm perches: 73.6 ± 12.8 s vs. 9.9 ± 15.7 s (p < 0.001). Butorphanol decreased latency to land in fractured hens (p ≤ 0.05).	KBD reduced perch-related mobility.
Nasr et al. (13)	Floor pen with perches; Lohmann Brown hens; 102 hens; 33–42 weeks; KBF assessed by palpation and dissection using a 0–3 scale.	Frequency of perch access; latency to fly down from perches.	Hens without fractures accessed 100-cm perches more frequently than hens with fractures (p = 0.016). Latency to fly down from 50-cm and 100-cm perches was longer in hens with fractures (P = 0.01 and p < 0.001, respectively).	KBD reduced perch access and perch-related mobility.
Nasr et al. (25)	Floor pen with raised perches; Lohmann Brown hens; 103 hens; 35–38 weeks; KBF assessed by palpation and dissection.	Latency to fly down from 50-, 100-, and 150-cm perches.	Birds with fractures took longer to fly down from all perch heights than birds without fractures (p < 0.05). From 150 cm, fractured birds took 11.5 ± 13.2 s vs. 4.2 ± 3.1 s in the carprofen group, and 100.3 ± 123.6 s vs. 3.0 ± 4.6 s in the meloxicam group.	KBD reduced perch-related mobility.
Casey-Trott and Widowski (27)	Furnished cages; LSL-Lite hens; 72 hens; 72 weeks; KBF assessed by palpation and confirmed by dissection; hens classified as F0, no fracture; F1, single fracture; F2, multiple fractures.	Perching duration; resting location on floor vs. perch.	Fracture severity affected perching duration (p = 0.0436) and resting location (p = 0.0161). F2 hens perched longer than F0 and F1 hens: 20.0 ± 2.9% vs. 11.6 ± 3.2% and 13.9 ± 3.6%. Floor resting declined from 80.0 ± 6.9% in F0 hens to 56.9 ± 12.4% in F1 hens and 51.5 ± 7.7% in F2 hens.	KBD increased perch resting and shifted resting location away from the floor.
Gebhardt-Henrich and Fröhlich (28)	Aviary-like pens; 80 focal white (Nick Chick) and brown (Brown Nick) hens; 18–65 weeks of age; longitudinal monitoring of spontaneous fracture development via 43 repeated palpations (Score 1: severe to 4: normal).	Night perching; nest use after new fracture.	Hens with new fractures spent more time in the nest during the 10 d after fracture than during the 10 d before fracture (P = 0.01). Almost all hens perched at night regardless of fracture status.	Night perching was maintained; KBD changed non-perch resting/hiding behaviour.
Leblanc et al. (51)	Experimental static and swaying perches; LSL hens; 123 observations; 61 weeks; KBD assessed by palpation.	Latency to leave perch; perching duration under challenge.	Birds with keel damage remained longer on static perches than birds with foot lesions: 30.6 ± 2.7 s vs. 27.7 ± 2.7 s. No significant difference was found between keel-damaged and healthy birds in time taken to leave the perch under non-challenged conditions.	No clear perch-use association.
Ali et al. (30)	Commercial-style aviary; DeKalb White, Hy-Line W36, Bovans Brown, and Hy-Line Brown; 2,304 hens; 28, 54, and 72 weeks; keel fractures scored visually as 0/1.	Upper-tier use and upper-tier perch roosting at night.	Occupying the upper tier at night increased the odds of keel fracture by 5.4 times (p < 0.05). Roosting on the upper-tier perch specifically was associated with higher fracture odds (OR = 2.66).	Upper-tier perch roosting associated with greater KBD risk.
Baker et al. (21)	Enriched colony cages; Hy-Line W36 hens; 120 focal hens; 52–60 and 74–83 weeks; keel impacts measured with tri-axial accelerometers fixed at the keel.	Perch collisions, especially during ascent.	Collisions accounted for 81% of acceleration events ≥20 G-units. Most collisions were with the perch and occurred mainly while hens attempted to ascend onto it.	Perch interactions increased keel-impact risk.
Baker et al. (1)	Furnished cages; Hy-Line W36 hens; 120 focal hens; 52–83 weeks; KBD assessed by CT scans using F0–F2 scale.	Perch collisions and movement onto, off, or between perches.	Hens prone to collisions sustained more additional fractures over a 3-week period (p = 0.03). Collisions were most commonly associated with movement onto, off, or between perches.	Perch-related collisions increased KBD risk.
Pickel et al. (5)	Experimental cages; Lohmann White and Lohmann Brown hens; 36 hens; night-time pressure sensor film used on different perch designs.	Keel peak force and contact area while perching.	Pressure peaks on the keel were approximately 5 times higher than on a single foot pad. Square perches had lower peak force (p < 0.05) and larger contact area (p < 0.001) than round or oval perches. Soft prototype perches had lower peak force and larger contact area than all commercial perches (P < 0.001).	Perch design altered keel loading risk.
Rufener et al. (14)	Experimental platform-to-perch transitions; Nick Chick and Brown Nick hens; 40 hens; 27–28 weeks; only normal-keel hens tested by palpation.	Peak force at keel during landing on perches; balancing movements.	Long distances of 100 cm and steep angles increased peak forces and impulse at the keel during landing (P < 0.05). Hens were more likely to perform balancing movements after steep or long transitions.	Perch position increased mechanical risk at the keel.
DePaoli et al. (33)	Non-cage pens; four commercial and two random-bred strains; 120 hens; 18, 29, and 70 weeks; KBD assessed post-mortem by digital imaging using a 1–5 scale.	Perching biomechanics, especially up-down keel movement.	Up-down keel motion was positively correlated with number of deviations (r = 0.2, P < 0.001) and severity of deviations (r = 0.3, P < 0.001).	Perching biomechanics associated with KBD severity.
DePaoli et al. (46)	Enriched battery cages; Lohmann White and Lohmann Brown hens; 144 hens; 2–16 months; KBD assessed post-mortem by digital imaging using a 1–5 scale.	Perch shape and rocking motion while perching.	Round perches led to more severe KBD than mushroom perches (P < 0.05). Deviation severity was positively correlated with forward-backward keel rocking motion (r = 0.24, P < 0.001).	Perch shape and instability associated with KBD risk.
Donaldson et al. (52)	Commercial free-range houses; Hy-Line Brown hens; five farms, approximately 40,000 birds; 17–72 weeks; keel injuries assessed by palpation and dissection.	Access to aerial perches.	Aerial perch access did not significantly affect average palpated keel score (p > 0.05). However, treatment × farm interaction was significant (p < 0.05), with higher probability of high damage scores in the perched treatment on some farms.	Mixed evidence for aerial perch access.
Heerkens et al. (35)	Commercial aviaries, row vs. portal systems; 47 flocks; 60 weeks; KBD assessed by palpation for wounds, fractures, and deviations.	Perch vs. platform in front of nest box.	Keel wounds were less prevalent when a perch rather than a platform was provided in front of the nest box: 20.4% vs. 29.7% (p = 0.006).	Perch design reduced keel wound risk.
Heerkens et al. (37)	Aviary pens; ISA Brown and Dekalb White hens; 400 birds; 17–52 weeks; KBD assessed by palpation for fractures and deviations.	Ramps for accessing perches and nest boxes.	Ramps reduced keel bone fractures (F1, 950 = 45.80, p < 0.001) and mild deviations (p < 0.05) compared with pens without ramps.	Improved perch/elevated-resource accessibility reduced KBD risk.
Käppeli et al. (32)	Aviary/floor pens; LSL, LB, and LBPS hens; 8,000 hens; 6–65 weeks; keel deformities assessed by palpation using a 1–4 scale.	Perch material.	Rubber-coated metal perches were associated with a higher prevalence of moderate and severe keel deformities than plastic perches (p = 0.007).	Perch material influenced KBD risk.
Malchow et al. (53)	Floor pens with elevated structures; Lohmann Dual and Lohmann Tradition hens; 132 hens; 22–50 weeks; KBD assessed by radiography; perching assessed by scan sampling.	Elevated grid vs. perch use as resting structure.	Use of elevated structures was higher with grids than with perches (p < 0.001). Authors suggested grids may provide a larger contact area and may help reduce peak force on the keel.	Alternative elevated resting structure may reduce perch-related risk.
Scholz et al. (6)	Furnished cages, aviary, and small-group systems; four strains; 12 months; keel bones assessed macroscopically and histologically.	Pressure loading during perching.	Histology indicated that 40% of slight deviations occurred without fractures, consistent with adaptational remodelling from mechanical pressure during perching. Severe deformities were mainly linked to traumatic fractures from flight, landing accidents, or collisions.	Perching pressure associated with keel deviation.
Scholz et al. (34)	Experimental room; LSL, LT, and LB hens; 60 hens; 21 weeks; keel status assessed by palpation.	Safe vs. failed landings on different perch designs.	Safe landings were highest on prototype soft perches and lowest on round steel perches (p < 0.001). Safe landings declined above a 50-cm vertical jump threshold (p = 0.025).	Perch design influenced landing safety and potential KBD risk.
Sirovnik et al. (38)	Multi-tier aviary; Nick Chick and Brown Nick hens; 3,920 hens; 18–65 weeks; fractures assessed by palpation.	Feeding from perches vs. platforms; body instability.	Hens feeding from perches showed more body instability, including balancing and falls, than hens feeding from platforms (P < 0.001). Keel fracture prevalence did not differ between treatments (p = 0.555).	Perch use increased instability, but not KBF in this study.
Stratmann et al. (36)	Aviary system; Dekalb White and ISA Brown hens; 4,500 hens; 18–64 weeks; keel status assessed by palpation as non-damaged, deviated, or fractured.	Soft vs. hard perch material.	Birds with non-damaged keel bones were more frequent with soft perches than hard perches: 55.1% vs. 39.8% (p < 0.0001). Assignment to deviation or fracture was 1.8 and 1.9 times more likely, respectively, in pens with hard perches (P < 0.0001).	Soft perch material reduced KBD risk.
Stratmann and Ringgenberg (54)	Rearing and laying pens; LSL and LB hens; 150 focal hens; 17–38 weeks; fracture severity assessed by palpation using a 0.0–10.0 scale.	Five perch types.	Fracture severity increased with age (p < 0.001), but perch type did not affect fracture severity (p = 0.472).	No clear perch-type effect on KBF severity.
Wilkins et al. (29)	67 commercial UK flocks in different systems; end-of-lay hens; KBF assessed by dissection; internal design and perch heights recorded.	Maximum accessible perch height.	Maximum accessible perch height above slats and litter was strongly correlated with percentage of birds with fractures: r2 = 0.4136, P < 0.001 above slats; r2 = 0.5966, P < 0.001 above litter. Mean severity was also influenced by perch height.	Higher accessible perch height associated with greater KBF risk.

#### 3.3.1 KBD as a potential influence on perch access, use, and resting location

Evidence that KBD influenced perch-related behaviour depended on whether studies measured access, duration, or resting location. Evidence of impaired access was relatively consistent. In floor pens, hens without fractures accessed 100-cm-high perches more frequently than hens with fractures (13). Fractured hens also took longer to leave elevated perches. In a study by Nasr et al. (12), hens with fractures took six times longer to fly down from 100-cm-high perches and eight times longer from 150-cm-high perches than hens without fractures. Similar effects were reported by Nasr et al. (25), in which fractured hens took longer to fly down from perches at all three tested heights. In non-cage experimental pens, hens with a visible fracture gap were also less likely to access a perch or elevated nest box (19). Together, these studies indicate that keel fractures can reduce hens’ ability or willingness to perform perch-related vertical transitions.

Perching duration did not show a consistent direction. In individually housed furnished cages, hens with keel fractures spent less time perching than hens without fractures (10.03 ± 0.46% versus (vs.) 13.07 ± 0.99% (26);). In contrast, Casey-Trott and Widowski (27) found that hens with multiple fractures spent more time perching than non-fractured hens in furnished cages (20.0 ± 2.9% vs. 11.6 ± 3.2%).

Resting-location results also varied with housing context. In furnished cages, fractured hens shifted their resting location away from the floor and toward the perch. Non-fractured hens spent 80.0 ± 6.9% of their resting time on the floor, whereas hens with single and multiple fractures spent 56.9 ± 12.4% and 51.5 ± 7.7%, respectively (27). In aviary pens, Gebhardt-Henrich and Fröhlich (28) reported that hens with new fractures spent more time in the nest during the 10 days after fracture detection than during the preceding 10 days, while almost all hens continued to perch at night regardless of fracture status.

#### 3.3.2 Perch use and design as potential contributors to KBD

Evidence in the reverse direction concerned whether perch use, positioning, and design potentially contributed to KBD. Greater perch height and roosting at upper levels were associated with increased fracture risk. In commercial flocks, maximum accessible perch height was strongly correlated with the percentage of hens with fractures in areas above both slats and litter (29). In a commercial-style aviary, night-time use of the upper tier increased fracture odds 5.4-fold, while roosting specifically on the upper-tier perch was associated with greater fracture odds (OR = 2.66 (30);). Experimental work also showed that long and steep transitions to perches increased peak forces and impulse at the keel during landing (14).

Perch material and shape also influenced keel loading, landing stability, and KBD. Pickel et al. (5) found that pressure peaks on the keel were approximately five times higher than those on a single foot pad during perching, while square and soft prototype perches reduced peak force or increased contact area. Soft perches also increased the proportion of hens with undamaged keel bones compared with hard perches (55.1% vs. 39.8%) and reduced the likelihood of deviations or fractures (31). Other studies reported a higher prevalence of moderate and severe deformities on rubber-coated metal perches than on plastic perches (32), more severe KBD on round perches than on mushroom-shaped perches (33), and safer landings on prototype soft perches than on round steel perches (34).

The routes provided for reaching elevated resources were also relevant. Ramps and other modifications connecting housing levels reduced falls and collisions and were associated with fewer fractures or mild deviations compared with systems without these modifications (35, 36).

Overall, the evidence supported both directions of the relationship. Existing KBD was most consistently associated with impaired perch access and delayed descent, while findings for perching duration and resting location were mixed. Conversely, perch height, upper-level use, transition configuration, material, shape, and accessibility were associated with keel loading, landing stability, or KBD occurrence.

### 3.4 Nest, feeder, and drinker resource use in relation to KBD

Evidence linking KBD to nest, feeder, and drinker use was limited and varied across resource types (Table 4). This section focuses only on direct resource-use outcomes and resource-area design. Broader litter use, tier use, and vertical spatial distribution are considered separately.

**Table 4:** Nest, feeder, and drinker outcomes associated with KBD.

Resource	Study	Housing, birds and KBD assessment	Outcome variable	Key findings	Evidence pattern
Nest	Gebhardt-Henrich and Fröhlich (28)	Aviary; 80 focal hens; 18–65 weeks; palpation.	Nest occupancy after new fracture.	Hens spent longer in the nest after fracture detection, increasing from about 30 to 40 min during egg laying.	KBD changed nest occupancy.
Nest/elevated access	Rentsch et al. (19)	Non-cage pens; 80 hens; 37–39 weeks; radiography.	Access to elevated nest box or perch.	Access decreased when a visible fracture gap was present; estimated access fell from about 0.60 to 0.37. Nest and perch access were not separated.	KBD reduced elevated-resource access.
Nest	Nasr et al. (13)	Floor pen; 102 hens; 33–42 weeks; palpation and dissection.	Nesting behaviour.	No association between fracture severity and nesting behaviour (*p* = 0.666).	No clear association.
Nest	Wei et al. (26)	Furnished cages; 90 hens; 17–42 weeks; palpation and dissection.	Nesting behaviour.	No difference between fractured- and normal-keel hens: 8.07 ± 0.54% vs. 7.03 ± 0.60% of observation time.	No clear association.
Nest-area design	Heerkens et al. (37)	Commercial aviaries; 47 flocks; 60 weeks; palpation.	Perch vs. platform in front of nest box.	Keel wounds were lower with a perch than a platform: 20.4% vs. 29.7%.	Nest-area design affected keel wounds.
Feeder	Wei et al. (26)	Furnished cages; 90 hens; 17–42 weeks; palpation and dissection.	Feeding behaviour and feed intake.	Fractured hens spent less time feeding: 31.03 ± 1.31% vs. 35.21 ± 0.83%. Feed intake was also lower by about 2–4 g/hen/day across laying periods.	KBD reduced feeding behaviour/intake.
Feeder	Nasr et al. (13)	Floor pen; 102 hens; 33–42 weeks; palpation and dissection.	Eating behaviour.	No association between fracture severity and eating behaviour (*p* = 0.464).	No clear association.
Feeder design	Sirovnik et al. (38)	Aviary; 3,920 hens; 18–65 weeks; palpation.	Feeding from perches vs. platforms.	Perch-feeding increased instability and falls, but keel fracture prevalence did not differ from platform-feeding (*p* = 0.555).	Feeder design did not alter KBF prevalence.
Drinker	Nasr et al. (13)	Floor pen; 102 hens; 33–42 weeks; palpation and dissection.	Drinking behaviour.	Drinking declined with increasing fracture severity (*β* = −0.293, *p* = 0.026).	KBD reduced drinking behaviour.
Drinker	Wei et al. (26)	Furnished cages; 90 hens; 17–42 weeks; palpation and dissection.	Drinking behaviour.	No difference between fractured- and normal-keel hens: 169.75 ± 16.94 vs. 177.25 ± 9.92 sampled events.	No clear association.

Nest-related evidence was mixed. The clearest temporal finding came from Gebhardt-Henrich and Fröhlich (28), who reported that hens remained in the nest longer during egg laying after a new fracture was detected. Nest occupancy increased from approximately 30 min before fracture detection to approximately 40 min after detection, suggesting prolonged nest use rather than improved nest access. In contrast, Nasr et al. (13) found no association between fracture severity and nesting behaviour, while Wei et al. (26) found no difference in nesting behaviour between hens with and without keel fractures.

Evidence regarding nest access was more difficult to interpret because some studies combined nest access with access to other elevated resources. Rentsch et al. (19) found that hens with a visible fracture gap were less likely to access a combined elevated perch-or-nest resource, with estimated access decreasing from approximately 0.60 in hens without a visible fracture gap to approximately 0.37 in hens with a visible gap. However, because perch and nest access were combined, this finding should be interpreted as reduced access to elevated resources rather than as a nest-specific effect. Nest-area design may also be relevant. Heerkens et al. (37) found fewer keel wounds when a perch rather than a platform was provided in front of the nest box (20.4% vs. 29.7%), although this effect concerned keel wounds rather than fractures or deviations.

Evidence regarding feeder use and feed intake was also sparse. Wei et al. (26) provided the strongest indication that keel fractures may reduce feeding-related behaviour. Hens with keel fractures spent less time feeding than hens without fractures, with feeding reduced by approximately four percentage points of the total observation time. Daily feed intake was also consistently lower in fractured hens across all reported laying periods, although the absolute differences were small, at approximately 2–4 g/hen/day. In contrast, Nasr et al. (13) found no association between fracture severity and eating behaviour during morning observations. Sirovnik et al. (38) examined feeder design rather than the effect of KBD on feeding behaviour. Feeding from perches reduced some competitive behaviours at the feeder but increased instability and falls and did not alter keel fracture prevalence compared with feeding from platforms.

Evidence regarding drinker use was very limited and inconsistent. Nasr et al. (13) found that drinking behaviour declined as fracture severity increased, suggesting that KBD may reduce drinking activity in some contexts. However, Wei et al. (26) found no difference in drinking behaviour between hens with and without keel fractures.

Overall, the evidence suggests that KBD may alter nest occupancy and reduce feeding behaviour or feed intake, but findings were not consistent across studies or resource types.

### 3.5 Ground-level and outdoor space use in relation to KBD

Evidence linking KBD to ground-level and outdoor space use was limited and generally showed no consistent association (Table 5). Across individual-level studies, hens with and without KBD did not differ clearly in outdoor range duration, pophole exit frequency, ranging score, or distance travelled into the range. Using radio-frequency identification (RFID) tracking, Larsen et al. (39) found no association between keel condition and either ranging score or the maximum distance reached within the outdoor range. Bari et al. (40) similarly reported comparable levels of keel damage among indoor, low-ranging, and high-ranging hens. Kolakshyapati et al. (41) also found no significant association between range use and keel damage, although the study was based on a small sample. Using video-based observations, Wurtz et al. (42) similarly found that daily range duration and pophole exit frequency were not associated with keel fractures, deviations, or combined KBD.

**Table 5:** Ground-level and outdoor space-use outcomes associated with KBD.

Area category	Study	Housing, birds, and KBD assessment	Outcome variable	Key findings	Evidence pattern
Range and pophole	Richards et al. (20)	Commercial free-range; 1,100 ISA Brown hens; 25–70 weeks; palpation score 0–2	Pophole use as proxy for outdoor access	Higher keel score was associated with reduced pophole use (estimate = −0.07, *p* < 0.001).	KBD may reduce access to the range, but pophole use does not measure time outdoors.
Range	Larsen et al. (39)	Commercial free-range; 291 Hy-Line Brown hens; RFID tracking; palpation	Outdoor ranging score and range distance	No association between keel condition and ranging score or distance reached outdoors (*p* > 0.05).	No clear link between KBD and outdoor range use.
Range and pophole	Wurtz et al. (42)	Organic free-range; 120 focal hens; 18–38 weeks; video tracking and palpation	Range duration and pophole exits	Range duration and exit frequency were not associated with KBD; example result for medial fractures: *p* = 0.901.	No clear link, but the study ended before peak KBD prevalence.
Range	Bari et al. (40)	Free-range; 307 Hy-Line Brown hens; RFID from 25–64 weeks; post-mortem keel assessment and CT	Long-term individual range-use pattern	Indoor, low-range, and high-range hens did not differ in KBD (χ^2^ = 0.15, *p* = 0.93) or callus count (*p* = 0.27).	Strong evidence that range-use pattern did not explain KBD in this study.
Range	Kolakshyapati et al. (41)	Commercial free-range multi-tier aviary; 30 Lohmann Brown hens; 74 weeks; post-mortem keel scoring	Percentage of available days using the range	Range use was not associated with keel damage (*p* = 0.273).	No clear association, but evidence is limited by small sample size.
Range	Bestman et al. (43)	169 free-range/organic flocks; palpation; farmer-estimated % hens outside	Percentage of hens outside at flock level	KBD correlated with % hens outside in univariable analysis (r = 0.308, *p* < 0.001), but was not retained in the final model.	Supporting evidence only; the association was not robust after adjustment.
Litter and wintergarden	Rufener et al. (8)	Multi-tier aviary; 60 LB and 60 LSL focal hens; infrared tracking and radiographic KBF severity score 0–10	Litter-zone and wintergarden occupancy	In LB hens, litter-zone time declined from about 276 to 66 min/day with increasing KBF severity (*p* < 0.0001). Wintergarden use was not clearly KBF-driven.	KBD may reduce litter/lower-area use in aviaries, but this overlaps with vertical redistribution.
Litter and wintergarden	Montalcini et al. (44)	Multi-tier aviary with wintergarden; 376 hens; radiographic KBF severity score 0–10	Space-use unevenness across zones including littered floor and wintergarden	KBF severity predicted reduced vertical movement, but did not clearly predict later changes in space-use behaviour.	KBD affected movement more than ground/outdoor space use.

One exception was Richards et al. (20), who found that higher keel scores were associated with reduced pophole use. This suggests that more severe keel damage may reduce access to the outdoor range, although pophole registrations are only a proxy for range access and do not show how long hens remained outside and their activity. At flock level, Bestman et al. (43) found a positive univariable association between the proportion of hens outdoors and KBD score, but KBD was not retained in the final multivariable model.

Evidence for litter and wintergarden use came mainly from aviary tracking studies and partly overlapped with broader vertical spatial-distribution outcomes. Rufener et al. (8) found that increasing KBF severity was associated with reduced litter-zone occupancy in Lohmann Brown hens, declining from approximately 276 to 66 min/day between the lowest and highest severity scores. However, wintergarden use was not clearly related to KBF severity and changed mainly with age. Montalcini et al. (44) similarly found that KBF severity affected movement behaviour, especially vertical distance travelled, but did not clearly predict later changes in overall space-use behaviour.

Overall, the available evidence showed no consistent association between KBD and outdoor range use, while findings for litter and wintergarden use were limited and suggested possible reductions in lower-area occupancy with increasing fracture severity.

## 4 Discussion

### 4.1 Synthesis of the main findings

This review shows that associations between KBD and the use of functional resources or housing areas are resource-specific rather than uniform. The evidence base was methodologically diverse but unevenly distributed, with a marked concentration on perches, elevated structures, and resting locations, while other functional resources and housing areas were much less frequently examined. The clearest pattern was associated with perches and other elevated structures. Hens with keel fractures accessed elevated resources less frequently, took longer to descend from perches, or performed fewer perch-related vertical transitions (12, 13, 19, 25, 26). In particular, hens with visible fracture gaps were less likely to access an elevated perch or nest-box area than hens without a visible gap (19). These findings indicate that fracture-related KBD is most consistently associated with resources whose use requires jumping, landing, balancing, or controlled descent.

Reduced access did not, however, consistently correspond to reduced occupancy. Wei et al. (26) found that fractured hens spent less time perching, whereas hens with multiple fractures in Casey-Trott and Widowski (27) perched longer and rested less on the floor than non-fractured hens. In addition, hens spent longer in the nest during the 10 days after a new fracture was detected than during the preceding 10 days (28). These findings are not necessarily contradictory. KBD may reduce the frequency or ease of reaching an elevated resource while increasing the time spent there once it has been reached. Prolonged occupancy should therefore not automatically be interpreted as unaffected access or stronger preference.

Evidence concerning nests, feeders, and drinkers was limited and inconsistent. Nesting behaviour did not differ according to keel status in Nasr et al. (13) or Wei et al. (26), although longer nest stays reported after fracture detection suggest that KBD may alter nest occupancy under some conditions (28). Similarly, Wei et al. (26) reported less feeding behaviour and slightly lower feed intake in fractured hens, whereas Nasr et al. (13) found no relationship between fracture severity and feeding behaviour. Drinking declined with increasing fracture severity in Nasr et al. (13), but no difference was detected by Wei et al. (26). The available evidence therefore does not support a consistent effect of KBD on drinking behaviour, and the small number of studies prevents firm conclusions about whether KBD compromises the use of these essential resources.

Associations with outdoor and ground-level areas were also generally weak. Most individual-level studies found no clear relationship between KBD and range duration, range-use category, pophole exits, or distance reached outdoors (e.g. (39–42),). A study by Richards et al. (20) was an exception, reporting reduced pophole use with increasing keel score. However, the predominance of null findings should be interpreted in relation to the outcomes measured. Broad indicators such as total range duration, range-use category, maximum distance reached, or pophole passage counts may not detect changes in latency to enter or leave, transition success, locomotor speed, route selection, spatial distribution within the range, or behaviours performed outdoors. KBD could therefore alter how hens access or use the range without producing a detectable change in these aggregated measures. Future studies should combine passage records with spatially resolved tracking and direct behavioural observation.

Evidence concerning litter and wintergarden areas was restricted to a small number of aviary studies. In Lohmann Brown hens, increasing fracture severity was associated with less time in the litter zone, whereas wintergarden use was not clearly related to fracture severity (8). Montalcini et al. (44) found that fracture severity predicted reduced subsequent vertical distance travelled but did not clearly predict later changes in overall area-use patterns. These findings suggest that the most detectable effects may be associated with movement between housing levels rather than avoidance of a particular ground-level resource.

Overall, fracture-related KBD was most consistently associated with access to and use of physically demanding elevated resources. The available evidence does not support an equivalent general reduction across all functional resources and housing areas. However, this pattern may reflect both a genuine functional relationship and the greater research attention given to perches and vertical movement.

### 4.2 Functional interpretation and welfare implications

The welfare significance of altered resource use depends on the dimension of use being measured. Access frequency, latency to reach or leave a resource, occupancy duration, and repeated use over time capture distinct aspects of the interaction between a hen and her environment and should not be treated as interchangeable outcomes. The contrasting patterns identified for perch access, perch occupancy, and nest duration indicate that occupancy cannot be used as a proxy for unrestricted access or preference. Longer stays may reflect a reduced willingness or ability to initiate another transition, although the underlying mechanism was not directly tested. Resource use should therefore be interpreted across three related components: access to the resource, occupancy of the resource, and the ability to leave it safely.

Pain is one plausible mechanism underlying these patterns. Keel fractures are considered painful, including after apparent healing (12, 45), and severe fractures may be associated with persistent negative affective consequences (22). Evidence from resource-related performance tests is nevertheless variable. Butorphanol reduced the time required for fractured hens to descend from elevated perches in Nasr et al. (12), whereas carprofen, meloxicam, and paracetamol did not consistently restore performance in other studies (19, 25). This variation may reflect differences in the analgesic agent, dose, treatment timing, fracture stage, or physical demands of the task.

Mechanical impairment may act alongside pain. The keel provides the attachment surface for the major flight muscles, which contribute to flight, landing, and wing-assisted transitions between levels (10, 11). Fractures may therefore interfere with balance, braking, landing, or controlled descent. The longer descent latencies, reduced vertical transitions, and lower vertical distances travelled by affected hens are consistent with this interpretation (12, 13, 19, 25, 44).

Behavioural compensation may further explain why KBD does not always result in complete resource avoidance. Hens may reduce the number of transitions, select less demanding routes, remain longer in one location, or redistribute themselves among housing levels. Almost all hens in Gebhardt-Henrich and Fröhlich (28) continued to perch at night after a new fracture was detected, suggesting that roosting motivation may persist even when access to or departure from the perch is uncomfortable or painful. Continued use of a highly valued resource should therefore not be interpreted as evidence that the hen is functionally unaffected.

The consequences of KBD are also likely to depend on resource design and placement. Large vertical gaps, long or steep transitions, unstable perches, and unsuitable landing areas increase the physical demands placed on hens. Long and steep transitions increased forces at the keel and the occurrence of balancing movements during landing, although these experiments were conducted in hens without KBD and therefore indicate injury risk rather than the consequences of established damage (14). Perch material, shape, and accessibility were also associated with keel loading, landing safety, deviations, or fractures in several studies (5, 31, 33, 34, 46). Ramps reduced fractures and mild deviations in experimental aviary pens, further indicating that the routes used to reach elevated resources are relevant to KBD risk (35).

These findings have two related welfare implications. First, elevated structures may contribute to the development of KBD when their design creates difficult transitions, unstable landings, or high keel loading. Second, the same resources may become more difficult to access after damage has occurred. Resource provision alone is therefore insufficient if affected hens cannot reach, use, or leave those resources safely and comfortably. Housing should preserve behavioural opportunities while reducing unnecessary physical demands through stable perches, suitable materials, intermediate platforms, ramps, smaller vertical gaps, and safer landing areas.

### 4.3 Methodological limitations and research priorities

Interpretation of the evidence was constrained by heterogeneity in the definition and assessment of KBD. Studies differed in hen age, strain, housing system, lesion type, severity, and assessment method. Palpation, radiography, computed tomography, and post-mortem examination also differ in their ability to detect fractures, deviations, healed lesions, fracture gaps, and displacement. Consequently, apparently similar KBD categories may not represent equivalent skeletal conditions across studies, particularly when fractures and deviations are combined within composite scores. Future longitudinal studies should therefore distinguish fractures from deviations and, where possible, report lesion stage, severity, anatomical location, displacement, callus formation, and visible fracture gaps (47). Repeated individual-level assessment would also help determine whether functional changes differ among new, healing, and healed lesions. Study selection was undertaken by one reviewer; hence, some risk of reviewer-related selection error cannot be excluded, although predefined eligibility criteria and a structured assessment process were applied at both screening stages to support consistent decisions.

A further limitation concerns the definition of resource use. Perch occupancy, descent latency, nest duration, pophole passage, outdoor duration, feeding behaviour, and zone occupancy represent different components of the interaction between hens and their environment. Perch occupancy does not demonstrate easy access, pophole registrations do not indicate total outdoor use, and litter-zone occupancy may reflect redistribution among housing levels rather than avoidance of litter. Future studies should therefore distinguish access frequency, access latency, occupancy duration, ability to leave, failed transitions, and repeated use over time. Similarly, pophole passage, time outdoors, distance from the house, and behaviour performed on the range should not be treated as equivalent outcomes.

Automated tracking provides detailed repeated records, but detection near a resource should not automatically be interpreted as actual resource use. RFID and related systems generally record the presence of tagged hens within predefined detection zones or their passage through monitored points. These records may not establish whether hens interacted with the resource, how they used it, or whether access was difficult (48). RFID pophole records, for example, quantify passages rather than total outdoor activity (20), while aviary tracking systems quantify zone occupancy and transitions without directly recording hesitation, instability, failed access, or difficulty leaving (8, 44). Combining automated tracking with video or focal observation would therefore provide a more valid assessment of functional resource access and use and help distinguish actual interaction from proximity, restricted movement, prolonged occupancy, or behavioural compensation (49).

Temporal ordering was another major limitation. Many studies assessed KBD and resource use concurrently or within the same observation period, making it difficult to determine whether KBD altered subsequent use, whether previous use or exposure contributed to damage, or whether both were influenced by housing and bird-level characteristics. Observation periods also varied considerably, ranging from brief behavioural tests or a few days of sampling to repeated or continuous monitoring over much of the laying cycle. This variation may affect the reported associations because keel fractures heal over several weeks; Richards et al. (50) reported that 80% of hens entering the study with new fractures showed fracture healing after 35 days during a 6-week radiographic monitoring period. Short observation periods may therefore capture more immediate effects, whereas longer periods may combine fresh, healing, and healed lesions or allow behavioural adaptation. Future studies should prioritise longitudinal designs that combine repeated KBD assessments with repeated or continuous measurements of resource and area use. Behaviour before and after the detection of a new fracture, as well as lagged relationships between earlier resource use and later KBD, should be analysed explicitly. Null findings should also be interpreted cautiously because they may reflect modest analytical samples, short observation periods, low lesion prevalence, or insufficient distinction among lesion types, severities, and healing stages.

Finally, the evidence base was strongly concentrated on perches and other elevated structures and represented a limited range of housing contexts. Individual-level analyses were also generally based on modest analytical samples. Nest, feeder, drinker, litter, wintergarden, and outdoor outcomes were examined much less often. The apparent sensitivity of elevated-resource outcomes may therefore reflect both a genuine functional relationship and the greater research attention given to these resources. Future work should broaden the range of resources examined and include intervention studies that evaluate whether housing modifications preserve safe resource use after KBD has occurred. Existing evidence indicates that perch material, shape, transition distance, and ramp provision can influence keel loading, landing safety, or KBD risk (5, 14, 31, 34, 35). The next step is to determine whether these modifications also help hens with established KBD reach and leave elevated resources safely. The placement and accessibility of nests, feeders, drinkers, and popholes warrant similar investigation because their use by affected hens remains poorly understood.

## 5 Conclusion

The relationship between KBD and the use of functional resources and housing areas is context-dependent. The most consistent evidence concerns fracture-related KBD and elevated resources, particularly perches and elevated nest areas, where affected hens showed reduced access, delayed descent, fewer vertical transitions, or altered occupancy. Evidence concerning nests, feeders, drinkers, outdoor ranges, litter, and wintergardens was less extensive and often inconsistent. Hens with KBD do not necessarily stop using resources. They may access them less frequently, remain there longer, redistribute among housing areas, or continue highly motivated behaviours despite pain or mechanical impairment. Resource occupancy alone should therefore not be interpreted as evidence of unrestricted access. The central welfare question is not simply whether resources are available or occupied, but whether hens with KBD can reach, use, and leave them safely, comfortably, and with meaningful choice. Housing design and welfare assessment should consequently consider both the risk that resource configuration contributes to KBD and the capacity of affected hens to continue using the environment after damage has occurred.
